# Enhancing Electrochemical Sensing through Molecular Engineering of Reduced Graphene Oxide–Solution Interfaces and Remote Floating-Gate FET Analysis

**DOI:** 10.1021/acsami.4c03999

**Published:** 2024-05-15

**Authors:** Wen Zhuang, Hyun-June Jang, Xiaoyu Sui, Byunghoon Ryu, Yuqin Wang, Haihui Pu, Junhong Chen

**Affiliations:** † Pritzker School of Molecular Engineering, University of Chicago, Chicago, Illinois 60637, United States; ‡ Chemical Sciences and Engineering Division, Physical Sciences and Engineering Directorate, Argonne National Laboratory, Lemont, Illinois 60439, United States

**Keywords:** surface engineering, surface charge density, analytical interface, reduced graphene oxide, FET sensor

## Abstract

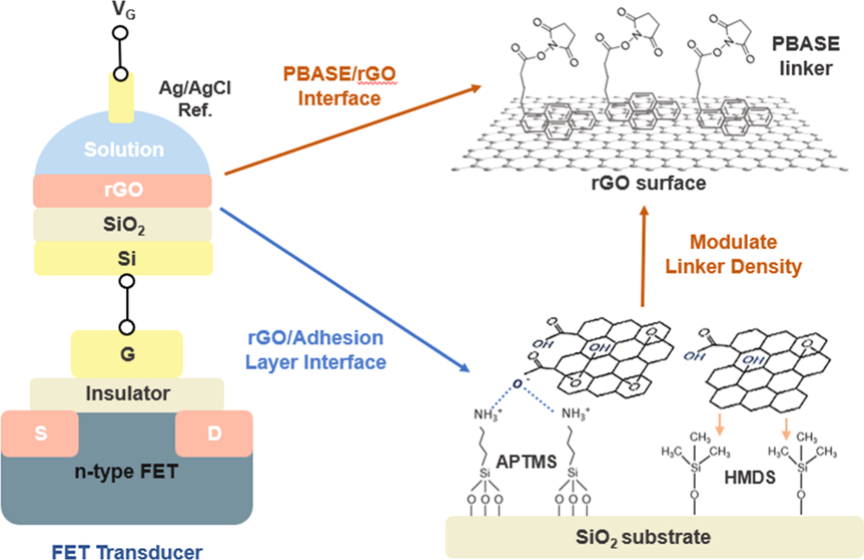

Two-dimensional nanomaterials such as reduced graphene oxide (rGO) have captured significant attention in the realm of field-effect transistor (FET) sensors due to their inherent high sensitivity and cost-effective manufacturing. Despite their attraction, a comprehensive understanding of rGO–solution interfaces (specifically, electrochemical interfacial properties influenced by linker molecules and surface chemistry) remains challenging, given the limited capability of analytical tools to directly measure intricate solution interface properties. In this study, we introduce an analytical tool designed to directly measure the surface charge density of the rGO–solution interface leveraging the remote floating-gate FET (RFGFET) platform. Our methodology involves characterizing the electrochemical properties of rGO, which are influenced by adhesion layers between SiO_2_ and rGO, such as (3-aminopropyl)trimethoxysilane (APTMS) and hexamethyldisilazane (HMDS). The hydrophilic nature of APTMS facilitates the acceptance of oxygen-rich rGO, resulting in a noteworthy pH sensitivity of 56.8 mV/pH at the rGO–solution interface. Conversely, hydrophobic HMDS significantly suppresses the pH sensitivity from the rGO–solution interface, attributed to the graphitic carbon-rich surface of rGO. Consequently, the carbon-rich surface facilitates a denser arrangement of 1-pyrenebutyric acid *N*-hydroxysuccinimide ester linkers for functionalizing capturing probes on rGO, resulting in an enhanced sensitivity of lead ions by 32% in our proof-of-concept test.

## Introduction

The growing demand for field-deployable, sensitive, and cost-effective sensors has spurred extensive research into nanomaterial-based field-effect transistor (FET) sensors over the recent decades. This interest is driven by their distinct advantages, encompassing real-time detection, lower detection limits, miniaturized system, and digital readouts.^1−3^ Two-dimensional (2D) nanomaterials, notably reduced graphene oxide (rGO),^4,5^ have emerged as pivotal components in various biosensor and electrochemical sensor applications. This is attributed to their exceptional mechanical, electrical, and other physicochemical properties, coupled with an easy fabrication process, scalable synthesis, and cost-effective production. Despite these promising attributes, there remain considerable challenges that must be addressed before achieving widespread commercial translation, particularly pertaining to the controllability of electrical and electrochemical properties of rGO for mass production.^6^

Addressing the challenges associated with the controllability of graphene oxide (GO) films during manufacturing is paramount. The inclusion of oxygen-containing functional groups, such as hydroxyl, epoxy, carbonyl, and carboxyl, on GO sheets serves to enhance solubility, promoting improved incorporation and uniform distribution within nanocomposites. Nevertheless, the presence of these oxygen functional groups introduces variability to the electrical properties of GO, a variability influenced by the chosen reduction methods and manufacturing parameters.^6^ In addition to these considerations, the manufacturing process can be further influenced by the surface energy of the substrate, encompassing factors such as hydrophobicity and hydrophilicity. This surface energy, in turn, impacts the orientation and density of GO flakes based on the substrate’s energy profile. To mitigate such variations, the utilization of self-assembled monolayers (SAMs) of (3-aminopropyl)trimethoxysilane (APTMS) and hexamethyldisilazane (HMDS) has gained widespread acceptance.^7^ This approach proves instrumental in enhancing the physical binding between rGO and the substrate.

Another critical concern arises at the solution interfaces on rGO, introducing additional unpredictable traits for rGO-based devices. Within the rGO film or adjacent materials, numerous ions present in the solution are free to drift under an electrical field. This phenomenon often triggers reduction reactions, resulting in nonuniform electrical properties of the rGO film. The extent of this nonuniformity is contingent upon the specific measurement conditions or the duration of exposure. Moreover, the choice of the linker for functionalizing biological or electrochemical probes on rGO, such as pyrenebutyric acid *N*-hydroxysuccinimide ester (PBASE),^8,9^ can introduce variations in rGO-based electronics. This variation stems from the imposition of different probe densities on rGO, consequently influencing the isoelectric points at the solution interface. These complexities underscore the need for a comprehensive understanding of the intricacies involved in solution interactions to design and optimize rGO-based devices effectively.

Despite the numerous adoptions of rGO–solution interfaces across various fields, including semiconducting devices,^10,11^ photocatalysts,^12,13^ and adsorbents,^14,15^ there is a lack of fundamental studies to understand the electrochemical interfacial properties and nonideal behaviors of rGO–solution interfaces caused by diverse variables aforementioned. Current analytical tools, such as X-ray photoelectron spectroscopy (XPS), Raman spectroscopy, and UV–vis spectroscopy,^16^ have limitations in directly characterizing the electrochemical interfaces of rGO for actual operational environments. Meanwhile, a remote floating-gate (RFG) FET platform^17−21^ offers a facile approach to overcome such challenges by capacitively connecting a sensing layer to the gate of the FET transducer. The target analytes on the sensing layer are electrically isolated by a dielectric layer and, thus, confined only to the solution interface. As a result, the RFGFET can monitor the changes of the intrinsic interfacial potential and assess pure electrochemical properties of solution interfaces with the following advantages: (1) prevention of redox reactions in testing interfacial materials due to the absence of current flow through them, (2) avoidance of effects related to interface traps and defects between targeted materials and substrates, (3) elimination of impedance influences from RFG modules, such as the thickness of the RG materials and SiO_2_, as well as the contact area of testing media on the RFG surface, and (4) enabling the characterization of intrinsic electrochemical properties of the solution interface without the effects described above.

**Figure 1:**
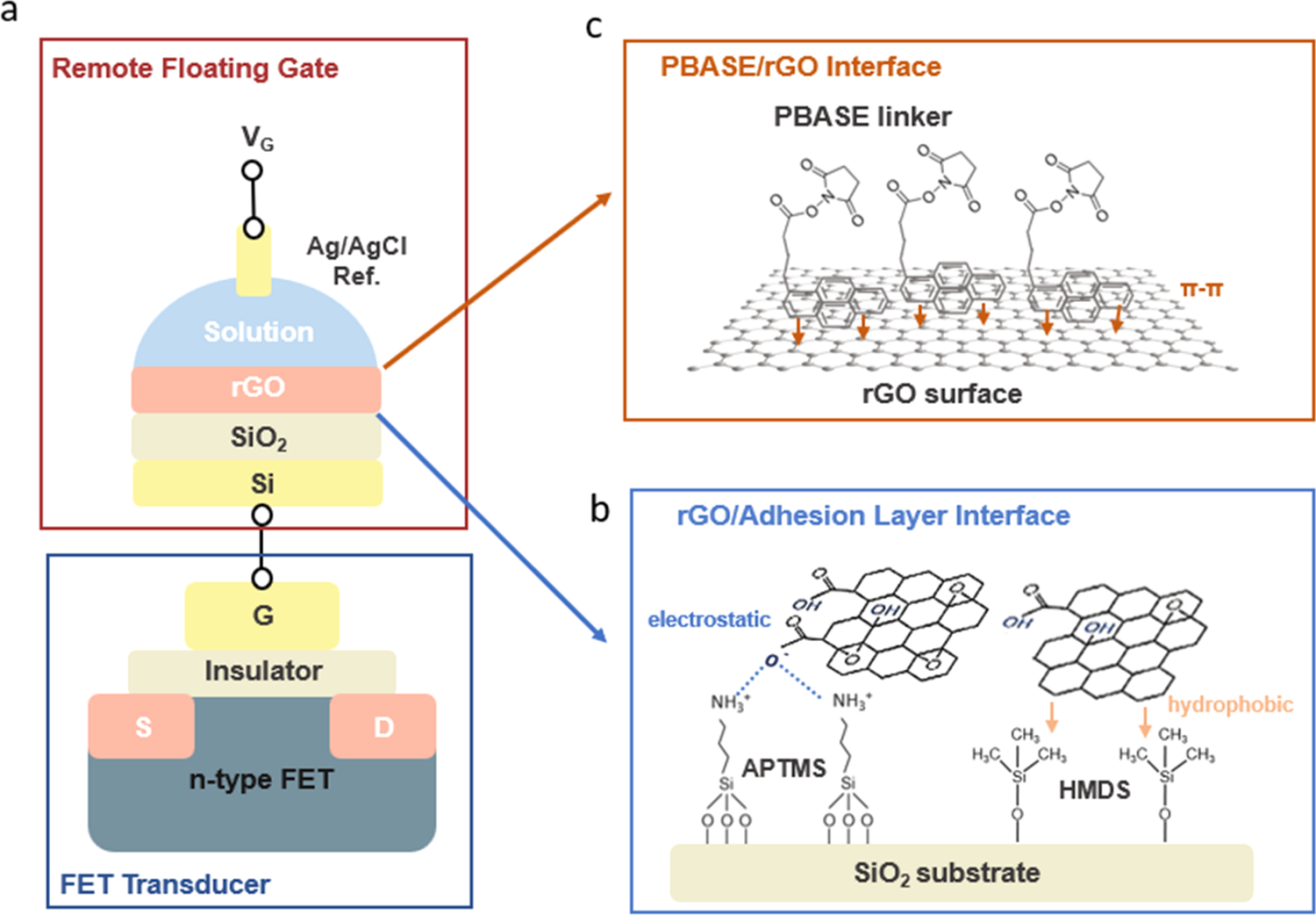
(a) Schematic illustration of the structure and configuration of RFGFET devices; (b) schematic illustration of the APTMS-treated and HMDS-treated substrate-rGO interface; and (c) schematic illustration of PBASE functionalization on the solution–rGO interface.

In this study, we developed an analytical method to characterize surface charge densities at the rGO–solution interface, leveraging the RFGFET platform (Figure 1). We observed that the surface charge density of the PBASE/rGO interface is highly influenced by the surface energy of adhesion layers between SiO_2_ and rGO, such as APTMS and HMDS. The HMDS/SiO_2_ substrate induces more hydrophobicity at the surface, attracting rGO with fewer oxygen functional groups during deposition. This observation is confirmed by XPS and the lower pH sensitivity of rGO/HMDS/SiO_2_ layers compared to that of rGO/APTMS/SiO_2_ layers, which exhibit more attachment of oxygen functional groups. Our analytical method also characterizes the surface charge density changed by the PBASE linker on top of the rGO surface. The results indicate that rGO/HMDS/SiO_2_ layers induce more binding sites for PBASE, resulting in a higher density of specific capturing probes for the analyte. As a proof of concept, rGO/HMDS/SiO_2_ increases the sensitivity of lead ions by 32% compared with those of rGO/APTMS/SiO_2_. This combination of analytical tools and surface engineering not only enhances our understanding of the electrochemical intricacies at the rGO–solution interface but also demonstrates practical improvements in the sensor performance, thereby showcasing its potential for broader applications in sensing technologies.

## Results and Discussion

### rGO/Adhesion Layer Interface

SAMs formed by two types of organosilicon molecules, APTMS and HMDS, were investigated to tailor the physicochemical properties of the interface and introduce additional interactions between the rGO thin film and the substrate. GO is an atomically thin sheet of conjugated carbon atoms with a large number of reactive functional groups, such as hydroxyl, carboxyl, and epoxide, and it was utilized as the precursor for the deposition of an rGO thin film. The self-assembled layers by APTMS consist of −NH_2_ tail groups (p*K*_a_ ∼10),^22^ which could be protonated to positively charged −NH_3_^+^ in the aqueous environment (when pH < 10) to electrostatically interact with negative charges in the functional groups of GO (e.g., deprotonated COOH). In contrast, HMDS was utilized to functionalize the substrate with methyl groups and promote interfacial adhesion via hydrophobic interaction with the sp^2^ graphitic structures of GO.

**Figure 2:**
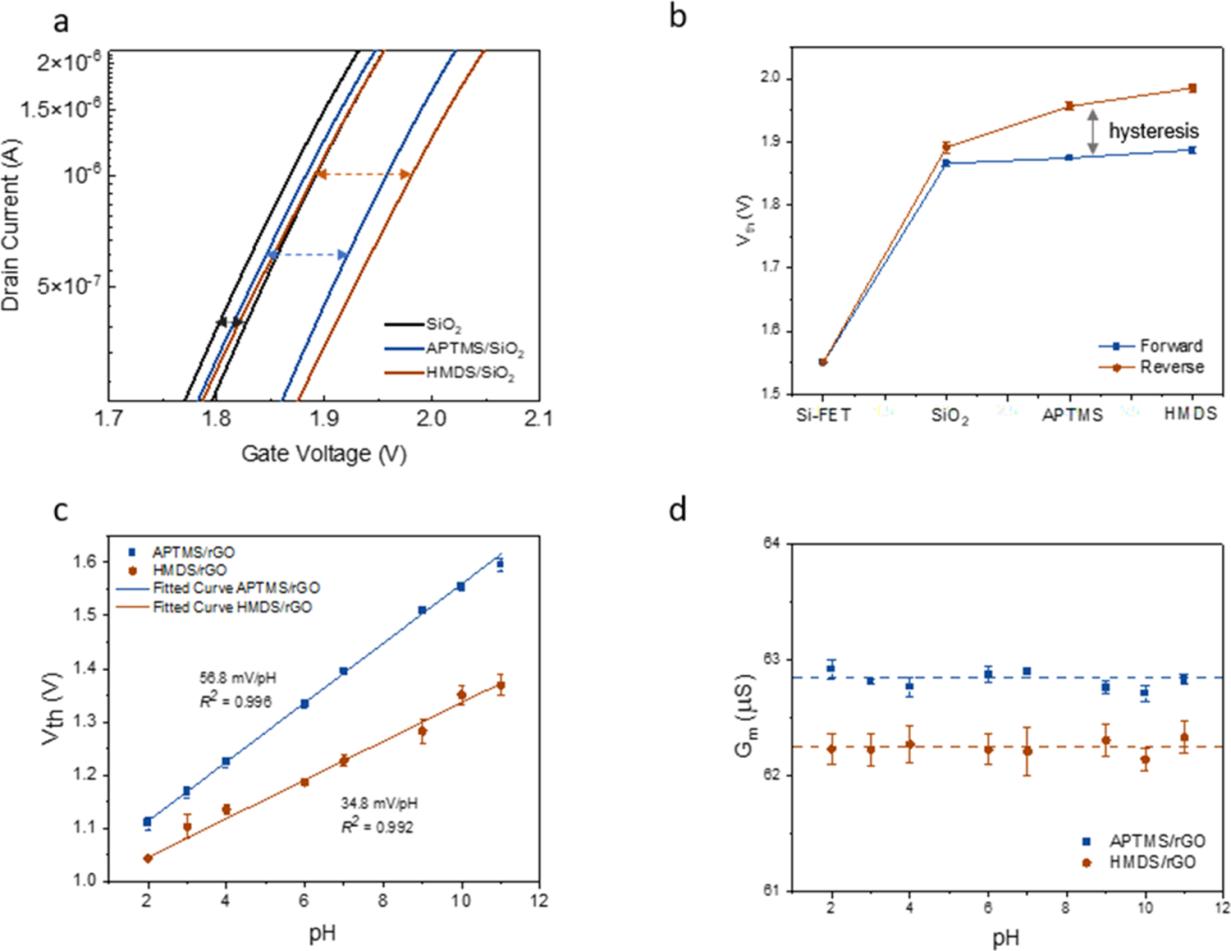
(a) RFGFET transfer curves of bare SiO_2_, APTMS/SiO_2_, and HMDS/SiO_2_ in pH 7; (b) *V*_th_ of Si-FET, RFGFET with RFG of bare SiO_2_, APTMS-treated SiO_2_, and HMDS-treated SiO_2_ in pH 7; (c) pH responses of rGO/APTMS/SiO_2_ and rGO/HMDS/SiO_2_; and (d) *G*_m_ of rGO/APTMS/SiO_2_ and rGO/HMDS/SiO_2_ vs pH.

The impacts of APTMS and HMDS without the rGO layer on the RFG were initially assessed by observing the transfer curve shifts of the RFGFET with different RFG configurations. These configurations included bare SiO_2_, APTMS/SiO_2_, and HMDS/SiO_2_ and were measured under a pH 7 buffer solution (Figures 2a and S1–S3). While the threshold voltages (*V*_th_) remained almost identical at forward sweeps, the *V*_th_ after surface treatments were significantly shifted up during the reverse sweeping mode, resulting in large hysteresis at the saturation regime (Figures 2b and S4). This increase in hysteresis may be attributed to the increased hydrophobicity caused by both APTMS and HMDS layers, which could slow down the orientational response of the water dipoles during the reverse sweeping mode.^20^ Additionally, HMDS treatment introduced larger hysteresis (98 mV) than APTMS treatment (82 mV), indicating that the methyl tail groups of HMDS could introduce more hydrophobic interfaces compared with the amine tail groups of APTMS.

Chemically modified substrates were then coated with GO thin films following the heat-assisted drop-casting method^17,18^ and postannealed under the same conditions. The prepared RFG surfaces were tested with various pH buffer solutions to evaluate their pH sensitivities from the oxygen functional groups. Devices based on both APTMS and HMDS exhibited stable responses during the entire measurement process and the detachment of the rGO thin film was seldom observed (Figure S5). As the pH value decreased from 11 to 2, both devices showed a decreasing trend in *V*_th_ (Figures 2c, S6 and S7), which resulted from the proton binding onto the residual oxygen functional groups of rGO thin films. However, they performed distinctly in terms of the pH sensitivity. rGO/APTMS/SiO_2_ presented a near-Nernstian sensitivity of 56.8 mV/pH, while rGO/HMDS/SiO_2_ possessed a limited pH sensitivity of 34.8 mV/pH, suggesting the potential selective attachment of more oxygen-rich rGO by APTMS. Moreover, as shown in Figure 2d, rGO/APTMS/SiO_2_ exhibited a larger transconductance (*G*_m_) than rGO/HMDS/SiO_2_, suggesting that rGO/APTMS/SiO_2_ exhibits a more stable solution interface with aqueous solutions.

Due to the self-limiting nature of the salinization reaction between APTMS/HMDS and the silicon-based substrate, APTMS and HMDS molecules can form a uniform self-assembled monolayer on SiO_2_ and thus functionalize the interface in a controllable manner. On the other hand, due to the high impedance of the FET chip, the bulk properties of the interlayer materials in the RFG module, such as thickness, density, and dielectric constant, would not influence the electrical characteristics of the sensor device. Instead, the interfacial properties including hydrophobicity, surface charge, and functional group play more important roles in determining the sensor performance.

**Figure 3:**
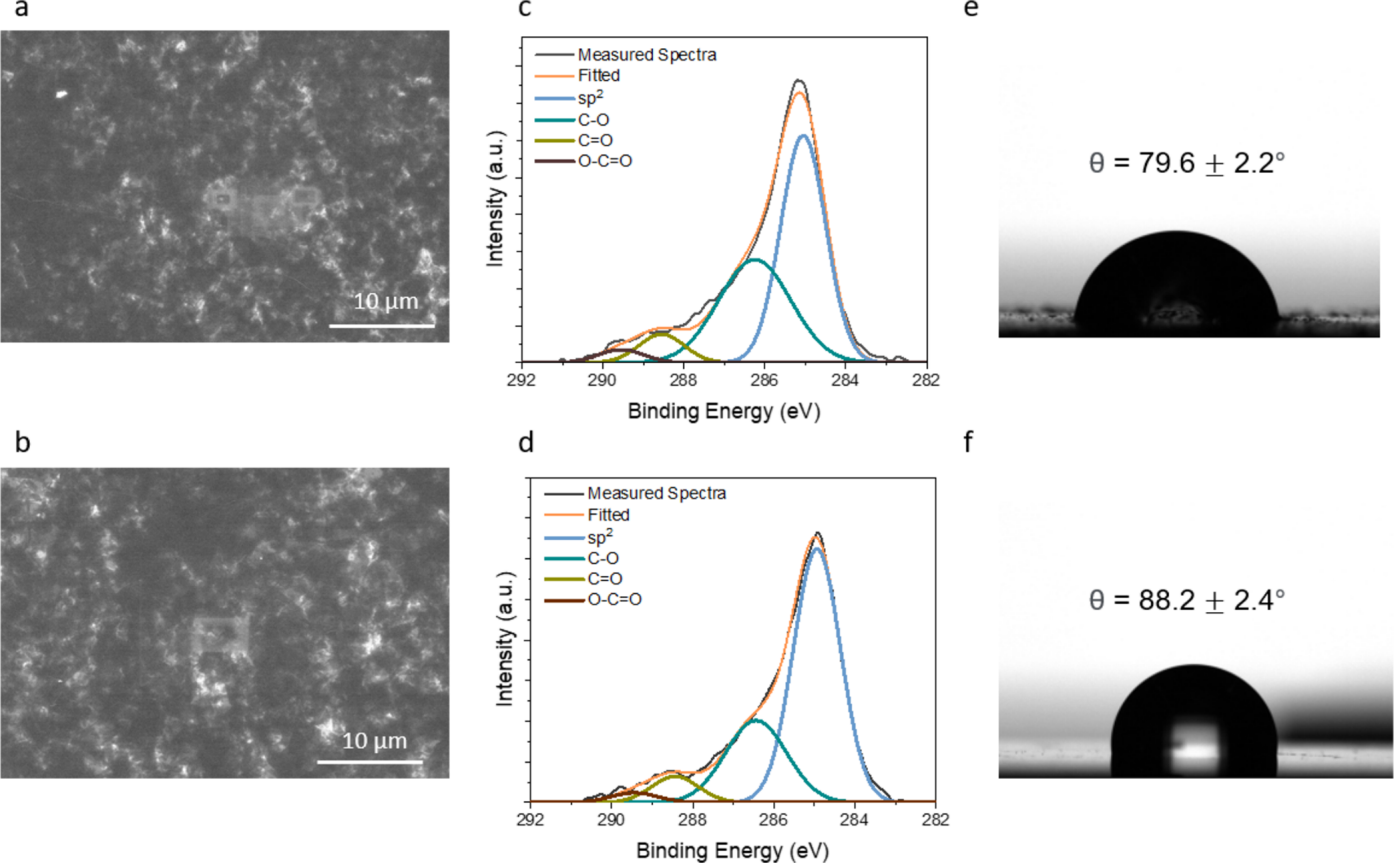
SEM images of (a) rGO/APTMS/SiO_2_ and (b) rGO/HMDS/SiO_2_. XPS spectra of (c) rGO/APTMS/SiO_2_ and (d) rGO/HMDS/SiO_2_. Contact angle measurements of (e) rGO/APTMS/SiO_2_ and (f) rGO/HMDS/SiO_2_.

Multiple characterization tools were then utilized to understand the mechanistic factors that caused the large difference in the pH sensitivity. SEM images in Figure 3a,b reveal that these devices share a similar surface morphology, i.e., a dense and continuous network of rGO nanoflakes with ca. 4 nm-thickness (measured by cross-sectional SEM and AFM^17,18^) covering the whole substrate. In contrast, the XPS spectra in Figure 3c,d reveal the difference in the chemical composition between rGO/APTMS/SiO_2_ and rGO/HMDS/SiO_2_. The deconvoluted C 1s region spectra for rGO/APTMS/SiO_2_ in Figure 3c show peaks with binding energies of 285.0, 286.3, 288.6, and 289.6 eV, which correspond to C=C (52.3 atom %), C–O (38.1 atom %), C=O (6.5 atom %), and O–C=O (3.1 atom %), respectively. However, for rGO/HMDS/SiO_2_, the deconvoluted C 1s peaks shown in Figure 3d with binding energies of 284.9, 286.4, 288.5, and 289.5 eV are assigned to C=C (64.2 atom %), C–O (26.8 atom %), C=O (6.5 atom %), and O–C=O (2.5 atom %), respectively. These XPS analyses suggest that rGO/APTMS/SiO_2_ contains a higher ratio of oxygen functional groups on the rGO surface, while rGO/HMDS/SiO_2_ contains a higher ratio of C=C bonds. Additionally, rGO/APTMS/SiO_2_ is more hydrophilic than rGO/HMDS/SiO_2_ according to contact angle analysis (Figure 3e,f). These observations suggest that APTMS/SiO_2_ can be deposited with rGO nanoflakes with larger amounts of oxygen functional groups via electrostatic interactions.^23^ Since both devices undergo an identical postannealing process, the results here indicate the possibility of the selective attachment of GO with more oxygen functional groups or graphitic carbon through surface treatment, which leads to a different chemical composition of rGO layers in the solution interface. With a higher ratio of oxygen functional groups on the rGO surface, rGO/APTMS/SiO_2_ possessed an increased number of active sites for proton binding, greatly contributing to the superior pH sensing capacity. In addition, the increased surface hydrophilicity caused by a higher ratio of oxygen functional groups could lower the activation energy and provide a favorable environment to promote proton binding, which further enhances the pH sensitivity of rGO/APTMS/SiO_2_.

### PBASE/rGO Interface

**Figure 4:**
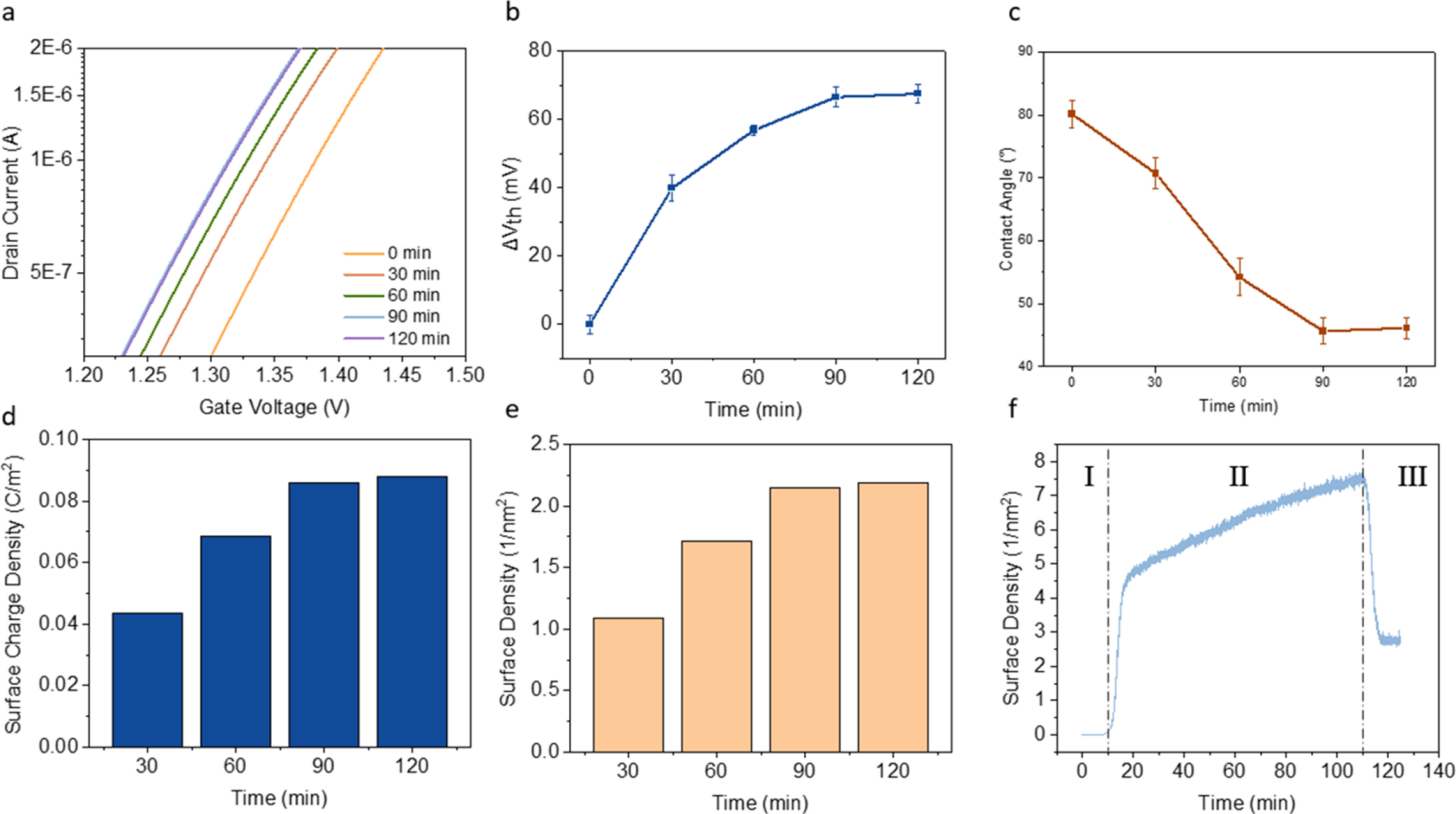
(a) Transfer curves of the RFGFET in pH 7 with increasing incubation time of PBASE on rGO/APTMS/SiO_2_. (b) Δ*V*_th_ in pH 7 with increasing incubation time of PBASE. (c) Contact angles vs incubation time of PBASE on rGO/APTMS/SiO_2_. (d) Calculated surface charge density and (e) surface density changed by increasing the incubation time of PBASE on rGO/APTMS/SiO_2_. (f) QCM measurement of PBASE adsorption on rGO/APTMS/SiO_2_.

We further characterized the influence of the PBASE linker on the rGO interface. As the incubation time of the PBASE linker on top of the rGO/APTMS/SiO_2_ surface increased, the threshold voltage (*V*_th_) of the transfer curves gradually decreased from 1.37 to 1.31 V (Figure 4a,b). These negative shifts suggested that the surface potential of the RFGFET sensors gradually decreased, potentially attributed to the charge transfer induced by the electron-withdrawing properties of the NHS group in PBASE molecules that led to a more positive potential on the rGO surface.^24^ Δ*V*_th_ saturated after 90 min of PBASE incubation, indicating the saturation of PBASE functionalization on the rGO surfaces. In addition, contact angle analysis of the rGO thin films revealed a similar trend of interfacial changes (Figures 4c and S8). PBASE steadily increased the hydrophilicity of the rGO surface, and the saturation point of hydrophilicity corresponds to the RFGFET results shown in Figure 4b.

According to the working mechanism of the RFGFET, the Δ*V*_th_ solely results from the alterations in the surface potential on the RFG. Assuming that the surface of the functionalized rGO is uniformly charged in the electrolytes, the surface charge density brought by PBASE functionalization could be modeled using the Poisson–Boltzmann theory and further calculated via the Grahame equation:
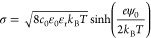
where σ is the surface charge density, *c*_0_ is the bulk concentration of electrolyte ions, ε_0_ is the permittivity of vacuum, ε_*r*_ is the relative permittivity of the medium, *k*_B_ is the Boltzmann constant, *T* is the temperature in Kelvin, and ψ_0_ is the surface potential. According to the results shown in Figure 4d, the surface charge density of PBASE at the solution–rGO interface increased rapidly within the first 90 min and reached a saturation point around 0.088 C/m^2^. In this model, the surface charge brought by PBASE functionalization is most likely originated from the charge transfer between rGO and PBASE, and we made a rough estimate based on previous studies that the average amount of charge per PBASE molecule on the interface is around 0.25 e (Supporting Note). Dividing the surface charge density by the average amount of charge per molecule, we finally had a quantitative estimate of the surface density of PBASE along the functionalization process (Figure 4e), which reached a saturated density of 2.20 molecule/nm^2^ at 90 min.

Furthermore, we conducted quartz crystal microbalance (QCM) measurements to verify the adsorption of PBASE on rGO and RFGFET characterization. A thin layer of rGO was deposited on the gold QCM chip and placed in the QCM chamber with a flow-cell setup. As shown in Figure 4f, the prepared sample was first stabilized with the dimethylformamide (DMF) solvent for 10 min (Phase I), exposed with a continuous flow of the PBASE/DMF solution for 100 min (Phase II) and finally washed with the DMF solvent to remove unbounded PBASE molecules (Phase III). This QCM measurement directly characterizes the mass change of the surface during the PBASE functionalization process and reveals a density of 2.82 PBASE molecules/nm^2^ on the rGO surface, which agrees well with the result of 2.20 molecule/nm^2^ by RFGFET sensor measurements.

**Figure 5:**
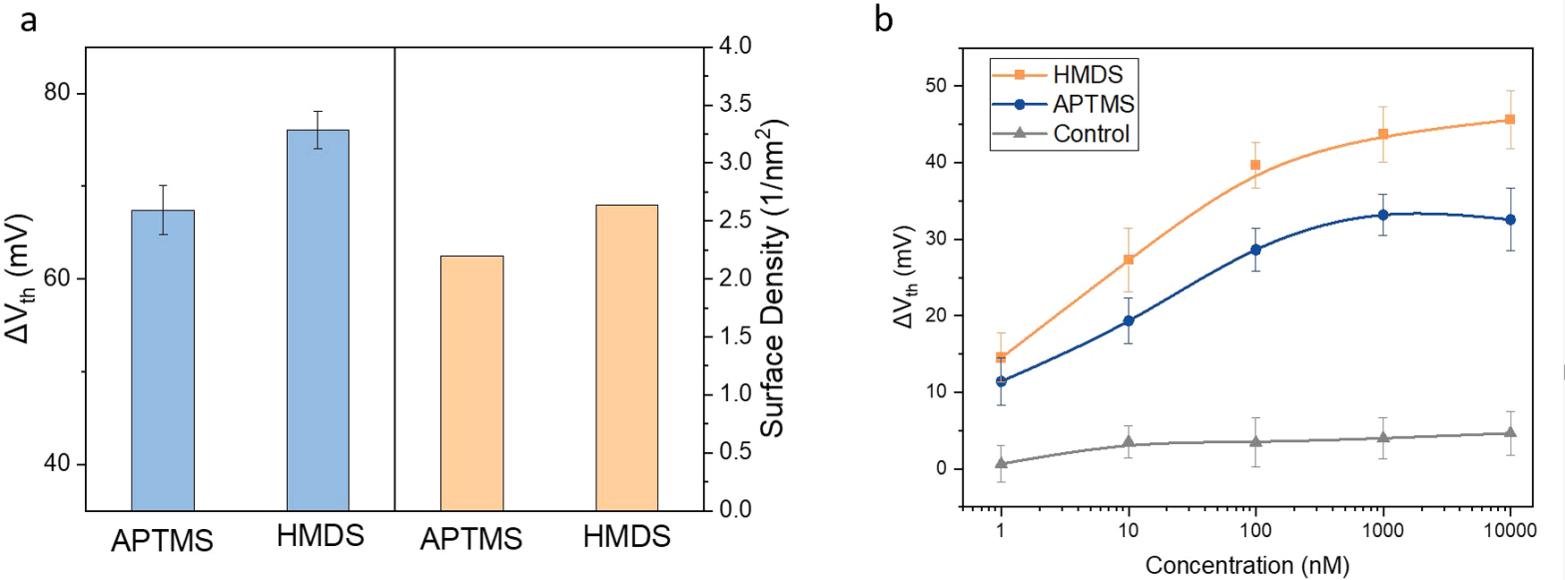
(a) Left: Δ*V*_th_ of rGO/APTMS/SiO_2_ and rGO/HMDS/SiO_2_ after PBASE incubation for 120 min and right: calculated surface density of PBASE from each case. (b) Lead ion sensitivity obtained from GSH-functionalized rGO/APTMS/SiO_2_, GSH-functionalized rGO/HMDS/SiO_2_, and nonfunctionalized rGO/APTMS/SiO_2_.

We further observed a higher PBASE density on rGO/HMDS/SiO_2_ than on rGO/APTMS/SiO_2_ (Figure 5a). The surface density of PBASE was calculated from Δ*V*_th_ of each RFG after PBASE incubation for 120 min. The calculated surface density of PBASE for rGO/HMDS/SiO_2_ is measured to be 2.65 molecules/nm^2^, which is increased by 20.5% compared with rGO/APTMS/SiO_2_. This improvement is likely caused by a higher fraction of graphitic carbon components for rGO/HMDS/SiO_2_, resulting in more opportunities for π–π stacking with PBASE molecules.

### Sensing Application

A higher density of the linker and the resulting increased density of specific capturing probes on solution interfaces typically lead to a higher sensitivity to the analyte. As a proof of concept, we functionalized glutathione (GSH) on rGO/APTMS/SiO_2_ and rGO/HMDS/SiO_2_, each incubated with PBASE (Figure 5b). GSH has been widely utilized in the field of lead ion detection due to its specific binding with lead ions and can be easily immobilized onto the graphene/rGO surfaces by forming an amide bond with the PBASE linker.^25−27^ Meanwhile, nonfunctionalized rGO/APTMS/SiO_2_ samples were prepared and included as the control group. Lead ion solutions with varying concentrations in a range of 1 nM to 10 μM were introduced onto each RFG. The sensor response for each concentration was recorded by measuring the transfer curve, and then Δ*V*_th_ compared to the baseline was extracted from each transfer curve. As shown in Figure 5b, Δ*V*_th_ of both GSH-functionalized devices exhibits an approximately linear relationship with the logarithm of concentration within the range of 1–1000 nM, reaching saturation upon 1,000 nM due to the fully occupied GSH probes by lead ions. In contrast, the control group generated negligible electrical responses to different concentration levels of lead ions, suggesting that the original rGO surface has almost no sensitivity toward lead ions. The sensitivity of rGO/APTMS/SiO_2_ was measured to be 6.1 mV/decade in the linear region, while rGO/HMDS/SiO_2_ showed a significantly higher sensitivity of 8.8 mV/decade due to the higher density of GSH probes and the PBASE linkers.

## Conclusions

In this study, we introduce a novel analytical tool, leveraging the RFGFET platform, to directly quantify the surface charge density at the rGO–solution interface. This approach offers valuable insights into the electrochemical interfacial properties influenced by linker molecules and surface chemistry. The study reveals that the hydrophilic nature of APTMS promotes the acceptance of oxygen-rich rGO, resulting in a superior pH sensitivity. Conversely, hydrophobic HMDS attracts rGO with more graphitic regions that suppress the pH sensitivity while enabling denser arrangements of PBASE linkers. The proof-of-concept testing with lead ions demonstrates a 32% enhancement in sensitivity, attributed to the increased density of GSH probes from the rGO/HMDS/SiO_2_ surface. This synergistic combination of our new analytical tools and interfacial engineering contributes to a profound understanding of the electrochemical intricacies at the solution interface of 2D nanomaterials, opening avenues for an improved sensor performance and expanded manufacturing applications across diverse domains.

## Experimental Section

### RFG Electrode Fabrication

The cleaned 4-in. silicon wafer with 300 nm-thick SiO_2_ was treated with an oxygen plasma for 5 min at 250 W under an O_2_ flow rate of 10 sccm in order to introduce hydroxyl groups on SiO_2_ surfaces. The wafer was fully immersed and incubated in a 5% APTMS (Sigma-Aldrich, 281778) or HMDS (Sigma-Aldrich, 440191) solution dissolved in ethanol (Sigma-Aldrich, 459836) for 2 h. After the surface was washed, the wafer was heated at 120 °C for 20 min. GO solutions of 0.24 mg/mL were prepared by dispersing GO (ACS Material, 7782-42-5) in deionized water aided by ultrasonication for 20 min. 16 mL of 0.24 mg/mL GO solution was drop-casted over the entire area of a 4-in. wafer and then baked at 120 °C for 1 h to obtain multilayer GO on the APTMS-treated or HMDS-treated SiO_2_ surface. The GO/APTMS/SiO_2_ and GO/HMDS/SiO_2_ wafers were sliced to 1 × 2 cm^2^ for the RFG module. The postannealing of the RFG module was performed using a horizontal furnace for 10 min under an argon gas environment, with a temperature of 200 °C.

### Surface Functionalization and Characterization

The fabricated rGO RFG electrodes were fully immersed in 10 mg/mL 1-pyrenebutyric acid *N*-hydroxysuccinimide ester (PBASE) (Santa Cruz Biotechnology, 114932-60-4) diluted in dimethylformamide (DMF) (Sigma-Aldrich, 227056) for 0, 30, 90, and 120 min, respectively. To prepare Pb^2+^ sensors, a 10 mM glutathione (GSH) (Sigma-Aldrich, G4251) aqueous solution was incubated on the rGO surface for 2 h after washing the rGO surfaces by DMF. The surface images were acquired by scanning electron microscopy (Carl Zeiss Merlin SEM). The chemical environment analysis of the rGO surface was accomplished through X-ray photoelectron spectroscopy (Kratos AXIS Nova). The contact angle analysis was performed on an optical tensiometer (Biolin Scientific Theta Flex). The measurement of PBASE adsorption on the rGO surface was conducted using a quartz crystal microbalance (QSense QCM-D).

### Electrical Measurement System

A commercial n-type MOSFET (CD4007UB) was used as a transducer to investigate the fabricated RFG module. A 20 μL testing media solution prepared above was placed on the RFG module. An Ag/AgCl reference electrode contacted the testing media solution in order to apply the gate bias in a range of 0–5 V for all measurements. All transfer curves were measured using a Keithley 4200A semiconductor analyzer with a drain voltage set at 50 mV, and the gate voltage was selected in the double-sweep mode. Transfer curves of the RGFET were repeatedly measured for 20 cycles under each testing media solution. The solution was removed by pipetting after each measurement. The *V*_th_ was calculated as the gate voltage corresponding to a drain current of 1 μA on each transfer curve. The *G*_m_ of each RGFET was calculated at its maximum value. Each Δ*V*_th_ point was obtained from the last point of Δ*V*_th_ in quasi-equilibrium for 20 measurements (5 min testing time) at each concentration.

## Supporting Information Available

Transfer curve measurements and threshold voltage analysis of SiO_2_, APTMS/SiO_2_, & HMDS/SiO_2_; hysteresis analysis; SEM characterization of rGO/APTMS/SiO_2_; transfer curve measurements of rGO/APTMS/SiO_2_ & rGO/HMDS/SiO_2_; contact angle measurements of PBASE functionalization; and estimation of the average charge carried by PBASE (PDF)
