# Supplementary material for: Enhancing Electrochemical Sensing through Molecular Engineering of Reduced Graphene Oxide–Solution Interfaces and Remote Floating-Gate FET Analysis
Source: ACS Appl Mater Interfaces. 2024 May 15;16(21):27961–8. doi: 10.1021/acsami.4c03999 (PMC11145583; doi:10.1021/acsami.4c03999)
Supplement: Supplementary file 1 — am4c03999_si_001.pdf [file am4c03999_si_001.pdf]

Supporting Information

Enhancing Electrochemical Sensing through Molecular Engineering of Reduced Graphene Oxide-Solution Interfaces and Remote Floating-Gate FET Analysis

Wen Zhuang, [†a, b] *Hyun-June Jang,* [†a, b] *Xiaoyu Sui,* [a, b] *Byunghoon Ryu,* [b] *Yuqin Wang* [a, b] *Haihui Pu* [a, b] *and Junhong Chen* [a,,b,*]

a. Pritzker School of Molecular Engineering, University of Chicago, Chicago, Illinois 60637, United States.

b. Chemical Sciences and Engineering Division, Physical Sciences and Engineering Directorate, Argonne National Laboratory, Lemont, Illinois 60439, United States.

† These authors contribute to the manuscript equally.

* Corresponding Author Email: junhongchen@uchicago.edu

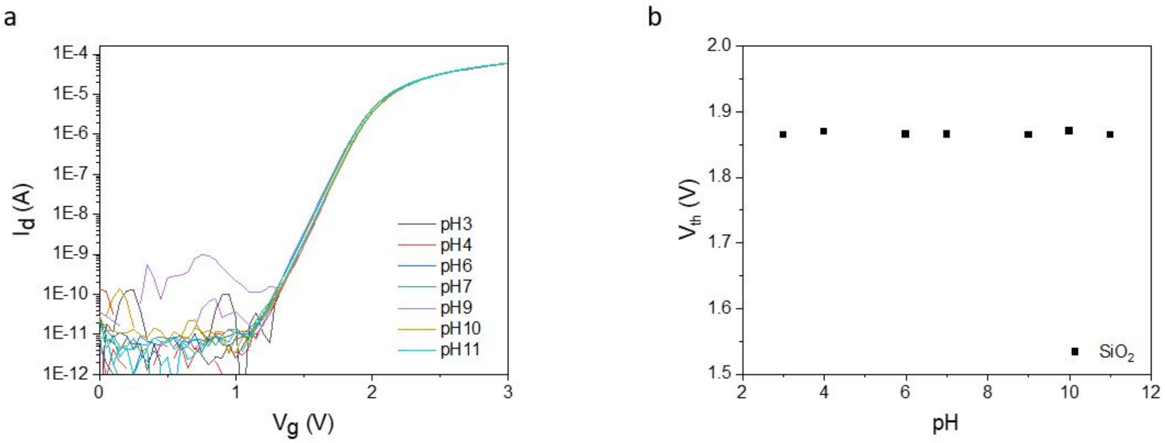

Figure S1: Transfer curves (a) and threshold voltages (b) of bare SiO_2 devices under different pH solutions.

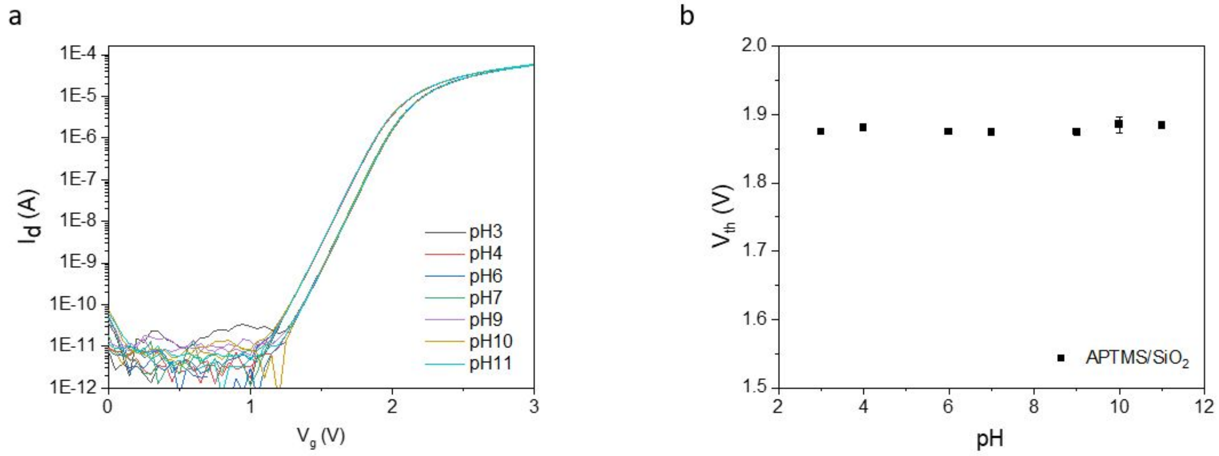

Figure S2: Transfer curves (a) and threshold voltages (b) of APTMS/SiO_2 devices under different pH solutions.

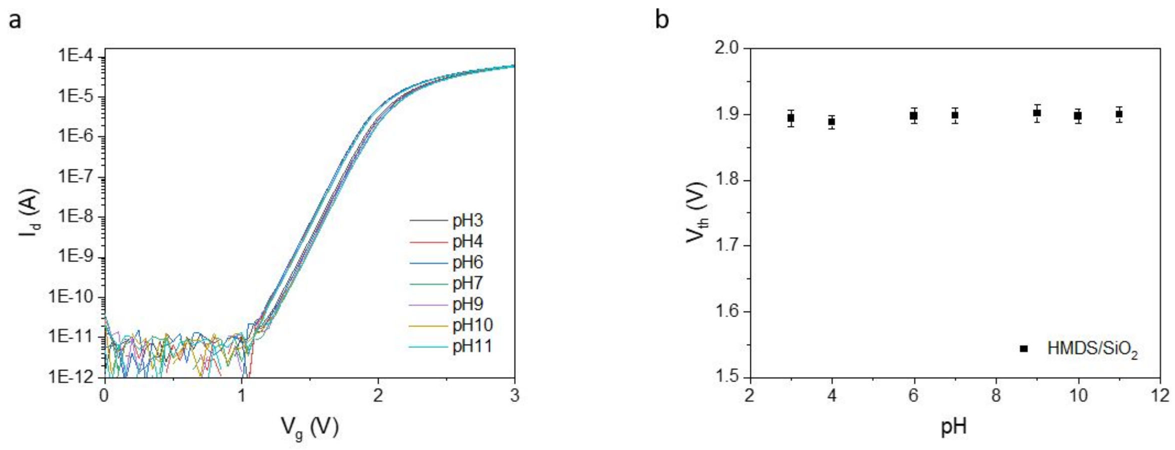

Figure S3: Transfer curves (a) and threshold voltages (b) of HMDS/SiO_2 devices under different pH solutions.

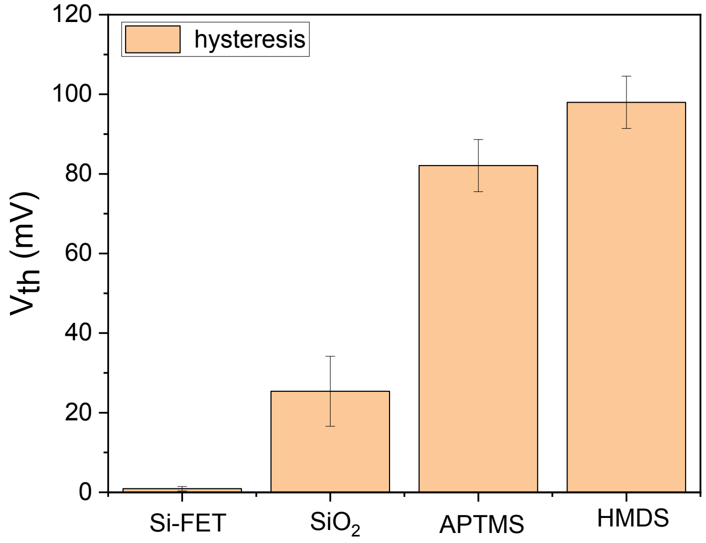

Figure S4: Calculated hysteresis of Si-FET, RFGFET with RFG of bare SiO_2, APTMS-treated SiO_2 and HMDS-treated SiO_2 in pH 7 PBS.

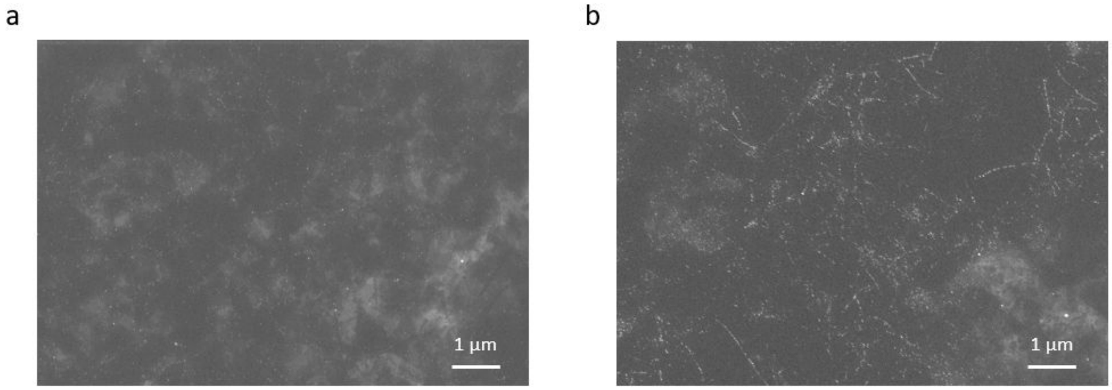

Figure S5: SEM images of rGO/APTMS/SiO_2 before testing (a) and after testing (b).

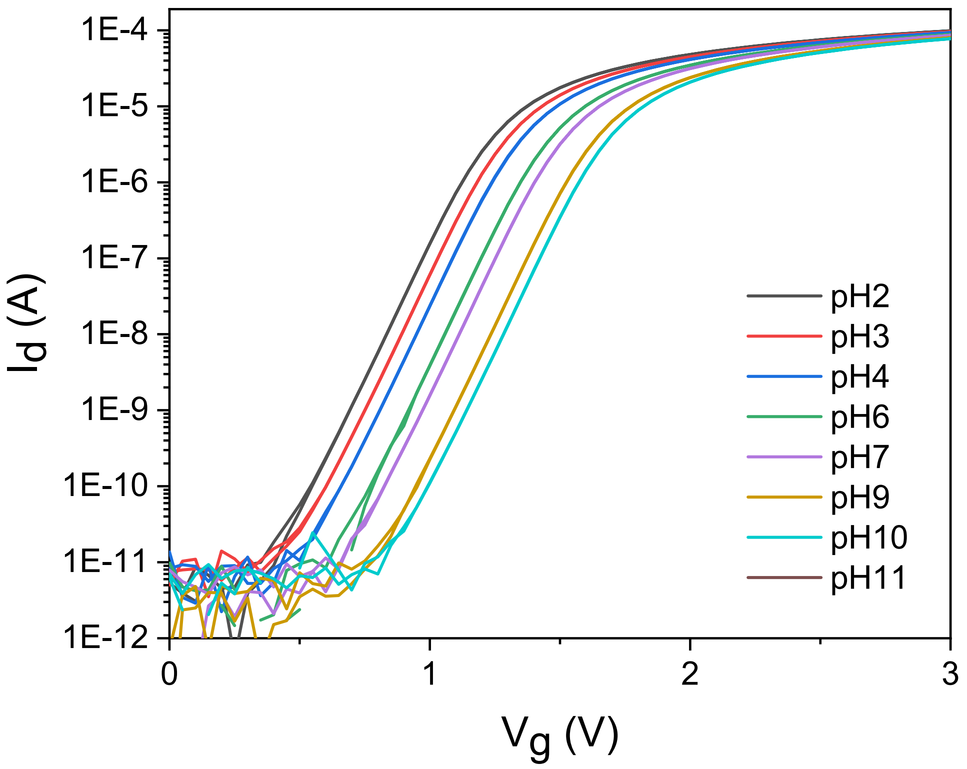

Figure S6: Transfer curves of rGO/APTMS/SiO_2 under different pH buffer solutions.

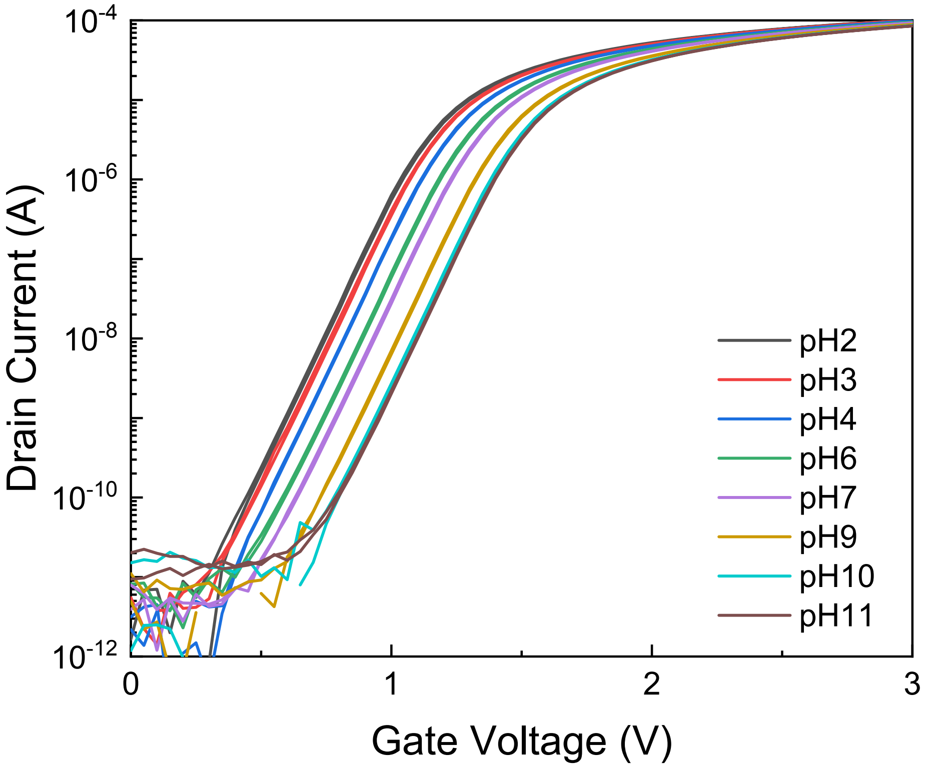

Figure S7: Transfer curves of rGO/HMDS/SiO_2 under different pH buffer solutions.

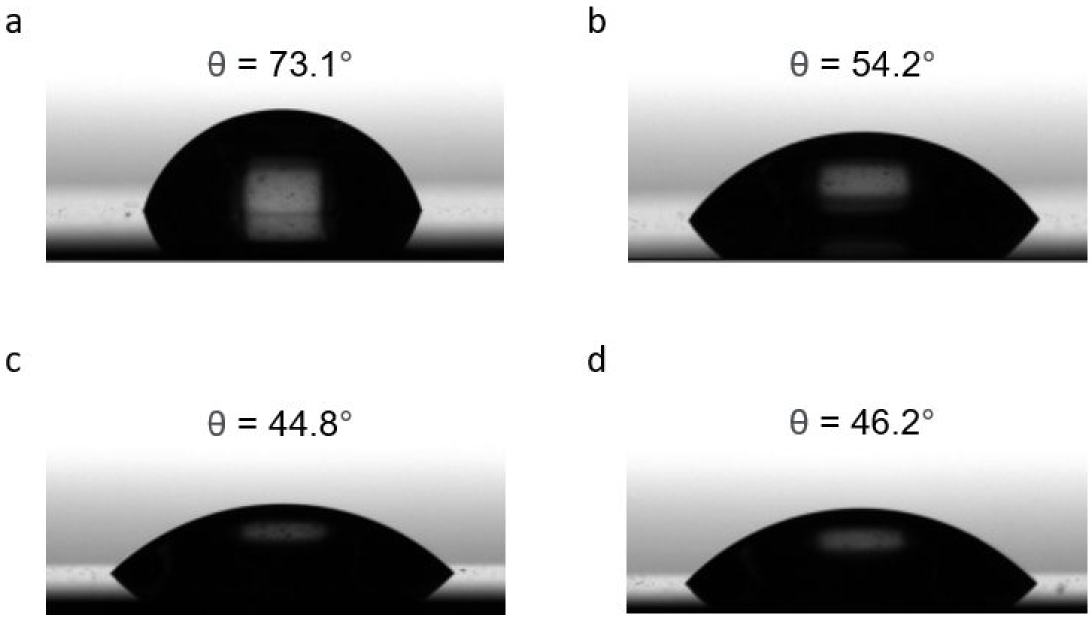

Figure S8: Contact angle measurements after 30-min (a), 60-min (b), 90-min (c) and 120-min (d) PBASE functionalization.

Supporting Note

Due to the electron-withdrawing nature of the NHS ester in the PBASE molecule, charge transfer will occur once the PBASE molecule binds onto rGO via π-π stacking. After the electron transfer from rGO to PBASE, the hole concentration in rGO will increase and PBASE will be negatively charged on the surface. Herein, we made a rough estimate of the average amount of charge each PBASE carries using experimental data from previous studies on graphene/PBASE interaction.

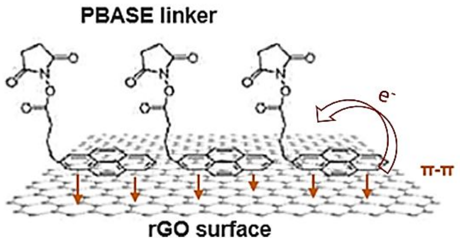

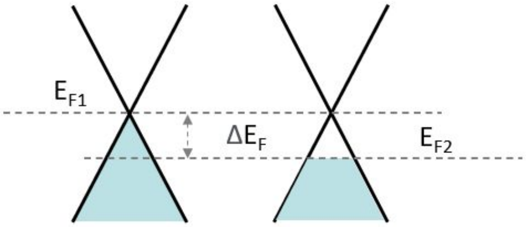

Liu et al. [1] measured the work function of graphene before and after the PBASE functionalization using Ultraviolet Photoelectron Spectroscopy (UPS), and concluded that the work function change brought by PBASE ($E_{Fi} - E_{Fp}$) is 0.171 eV. In graphene, the relationship between work function and carrier density:

$$n_h = n_i e^{(E_{Fi} - E_{Fp})/k_B T}$$

For graphene, the intrinsic carrier concentration at room temperature (300 K) is around $8.0 * 10^{10}$ cm^{-2}, so the hole density of PBASE-functionalized graphene can be calculated:

$$n_h = \sim 6.0 * 10^{13}\ cm^{-2}$$

Based on conservation of charge, the charge density of PBASE layer equals to the hole density of graphene:

$$n_e = \sim 6.0 * 10^{13}\ cm^{-2}$$

Mishyn et al. [2] studied the surface density of pyrene-based linkers on graphene using an electrochemical method, and we could obtain an estimated PBASE density over graphene using the same approach:

$$n_{PBASE} = \sim 2.4 * 10^{14}\ cm^{-2}$$

Therefore, we estimated the average charge each PBASE molecule carries:

$$\frac{n_e}{n_{PBASE}} = \sim 0.25\ e$$

References

1. Wu G, Tang X, Meyyappan M, Lai KW. Doping effects of surface functionalization on graphene with aromatic molecule and organic solvents. Applied Surface Science. 2017 Dec 15;425:713-21.
2. Mishyn V, Hugo A, Rodrigues T, Aspermair P, Happy H, Marques L, Hurot C, Othmen R, Bouchiat V, Boukherroub R, Knoll W. The holy grail of pyrene-based surface ligands on the sensitivity of graphene-based field effect transistors. Sensors & Diagnostics. 2022;1(2):235-44.
